# Statin Therapy for Hyperlipidemic Patients With Chronic Kidney Disease and End-Stage Renal Disease: A Retrospective Cohort Study Based on 925,418 Adults in Taiwan

**DOI:** 10.3389/fphar.2022.815882

**Published:** 2022-03-04

**Authors:** Fung-Chang Sung, Ying-Chin Jong, Chih-Hsin Muo, Chih-Cheng Hsu, Wen-Chen Tsai, Yueh-Han Hsu

**Affiliations:** ^1^ Department of Health Services Administration, China Medical University, Taichung, Taiwan; ^2^ Management Office for Health Data, China Medical University Hospital, Taichung, Taiwan; ^3^ Department of Food Nutrition and Health Biotechnology, Asia University, Taichung, Taiwan; ^4^ Division of Nephrology, Department of Internal Medicine, Ditmanson Medical Foundation Chia-Yi Christian Hospital, Chiayi, Taiwan; ^5^ Graduate Institute of Clinical Medical Science, College of Medicine, China Medical University, Taichung, Taiwan; ^6^ Institute of Population Health Sciences, National Health Research Institutes, Zhunan, Taiwan; ^7^ Department of Family Medicine, Min-Sheng General Hospital, Taoyuan, Taiwan; ^8^ Department of Medical Research, China Medical University Hospital, Taichung, Taiwan; ^9^ Department of Nursing, Min-Hwei Junior College of Health Care Management, Tainan, Taiwan

**Keywords:** cancer, chronic kidney disease, end-stage renal disease, heart disease, mortality, propensity-score, septicemia, statins

## Abstract

**Background:** For non-dialysis patients with hyperlipidemia, statins may provide clinical benefits in reducing mortality risk; however, the optimal treatment for dialysis patients with hyperlipidemia remains debatable. We evaluated the mortality risks for hyperlipidemic patients with renal disorders associated with statin therapy (ST), using the insurance claims data of Taiwan.

**Methods:** From hyperlipidemic patients diagnosed in 2000–2011, we identified 555,153 patients receiving statin treatment for at least 90 days continuously and 1,141,901 non-statin users, and then randomly selected, from both groups, the propensity score-matched subcohorts of statin users and nonusers in a 1:1 pair by renal function: 415,453 pairs with normal renal function , 43,632 pairs with chronic kidney disease (CKD), and 3,624 pairs with end-stage renal disease (ESRD). We compared the mortalities, by the end of 2016, from all causes, cancer, heart disease, and septicemia between statin users and non-users and between hydrophilic-statin users and lipophilic-statin users. The Cox method estimated ST users to non-user hazard ratios. The time-dependent model was also conducted as sensitivity analysis.

**Results:** The mean ages were 58.7 ± 10.7, 64.2 ± 10.7, and 62.2 ± 10.8 years in normal renal function, CKD, and ESRD groups, respectively. Compared with non-users, statin users had reduced mortality risks from all causes for 32%–38%, from cancer for 37%–46%, from heart disease for 6%–24%, and from septicemia for 17%–21% in all three renal groups. The hydrophilic statin therapy was superior than the lipophilic statin therapy, particularly for reducing deaths from all-causes and cancer. The results under the time-dependent model were similar.

**Conclusion:** Statin therapy is associated with reduced all-causes and non-cardiovascular mortality in ESRD patients.

## Introduction

The mortality is higher in dialysis patients than in the general population, approximately 16-fold higher for the US patients and 5-fold higher for Japanese patients (De Jager et al., 2009; Wakasugi et al., 2013). The main causes of deaths for patients with end-stage renal disease (ESRD) included cardiovascular disease (CVD), infection, cancer, and dialysis withdrawal (Den Hoedt et al., 2013; Vogelzang et al., 2015; Steenkamp et al., 2018). CVD has been the leading cause of deaths. However, the UK Renal Registry Annual Report indicated that the deaths from CVD decreased from 34% to 24% in 2000–2015 in adult dialysis patients (Steenkamp et al., 2018). The proportion of non-cardiovascular deaths for ESRD patients was increasing, especially in the elderly group (Den Hoedt et al., 2013; Vogelzang et al., 2015). The study from the ERA-EDTA registry showed an 82-fold increase in infection-related mortality and a near 3-fold increase in cancer-related mortality for dialysis patients compared to the general population (Vogelzang et al., 2015).

Statins are the first-line lipid-lowering medications for the patients of chronic kidney disease (CKD) with hyperlipidemia. Statin therapy (ST) could reduce deaths and CVD for 20% in CKD patients not requiring dialysis (Palmer et al., 2014). However, the optimal treatment for dialysis patients with hyperlipidemia remains inconclusive. For hemodialysis (HD) patients, Manson et al. reported that ST could reduce the mortality risks from all causes for 31%, from cardiac disease for 23%, and from noncardiac diseases for 44% (Fellstrom et al., 2009). In two randomized control trials, the AURORA study and the 4D (Die Deutsche Diabetes Dialyse) study, focusing on dialysis cohorts, found that rosuvastatin and atorvastatin could effectively reduce the serum levels of low-density lipoprotein cholesterol. However, both studies failed to observe the clinical benefits in reducing CVD mortality, all-cause mortality (ACM), and cardiovascular events (Wanner et al., 2005; Fellstrom et al., 2009). A meta-analysis has commented that ST contributed little benefits on mortality in dialysis adults (Palmer et al., 2013). In the *post-hoc* analyses of these two trials, the AURORA investigators remodeled the study design and found a 32% risk reduction from cardiac events in those with rosuvastatin treatment for patients with diabetes alone (Holdaas et al., 2011). For diabetic dialysis patients with pretreatment LDL-cholesterol >145 mg/dl in the 4D trial, März et al. reported that atorvastatin treatment could reduce 28%–52% of the risk for composite cardiovascular outcomes, cardiac deaths, and all-cause deaths (März et al., 2011). Recent studies using real-world data showed that ST could reduce the ACM for dialysis patients, particularly for HD patients (De Rango et al., 2016; Chung et al., 2017; Kim et al., 2018; Ota et al., 2019). A recent Korean population-based study found that ST was associated with reduced ACM for over 41% in HD patients (Jung et al., 2020).

Previous studies rarely used population-based data to examine simultaneously the effectiveness of ST associated with deaths from different causes for hyperlipidemia patients with renal disorders. We, therefore, used insurance claims data to investigate the effectiveness of ST in reducing the death risks not only from all causes but also from cancer, heart disease, and septicemia, for hyperlipidemic patients with kidney disorders, including patients with CKD and ESRD, compared with hyperlipidemic patients with normal renal function (NRF). Furthermore, the effectiveness between using hydrophilic statins (HSs) and lipophilic statins (LSs) was compared.

## Materials and Methods

### Data Source

We obtained the data from the Ministry of Health and Welfare Health and Welfare Data Science Center database, consisting of the National Health Insurance Research database (NHIRD) and the death registry of all insured people in Taiwan. NHIRD was composed of the information on the medical records of outpatient and inpatient claims for all residents in Taiwan. The death registry was composed of the information on the demographic status and cause of death of each deceased. These data sets were linked by recoded identifications to protect the privacy of the insured population. Drug and disease classifications conformed to the Anatomical Therapeutic Chemical (ATC) Classification System and the International Statistical Classification of Diseases and Related Health Problems [9th Revision (ICD-9) before 2016 and 10th Revision (ICD-10) since 2016].

#### Study Population Selection

**FIGURE. 1:**
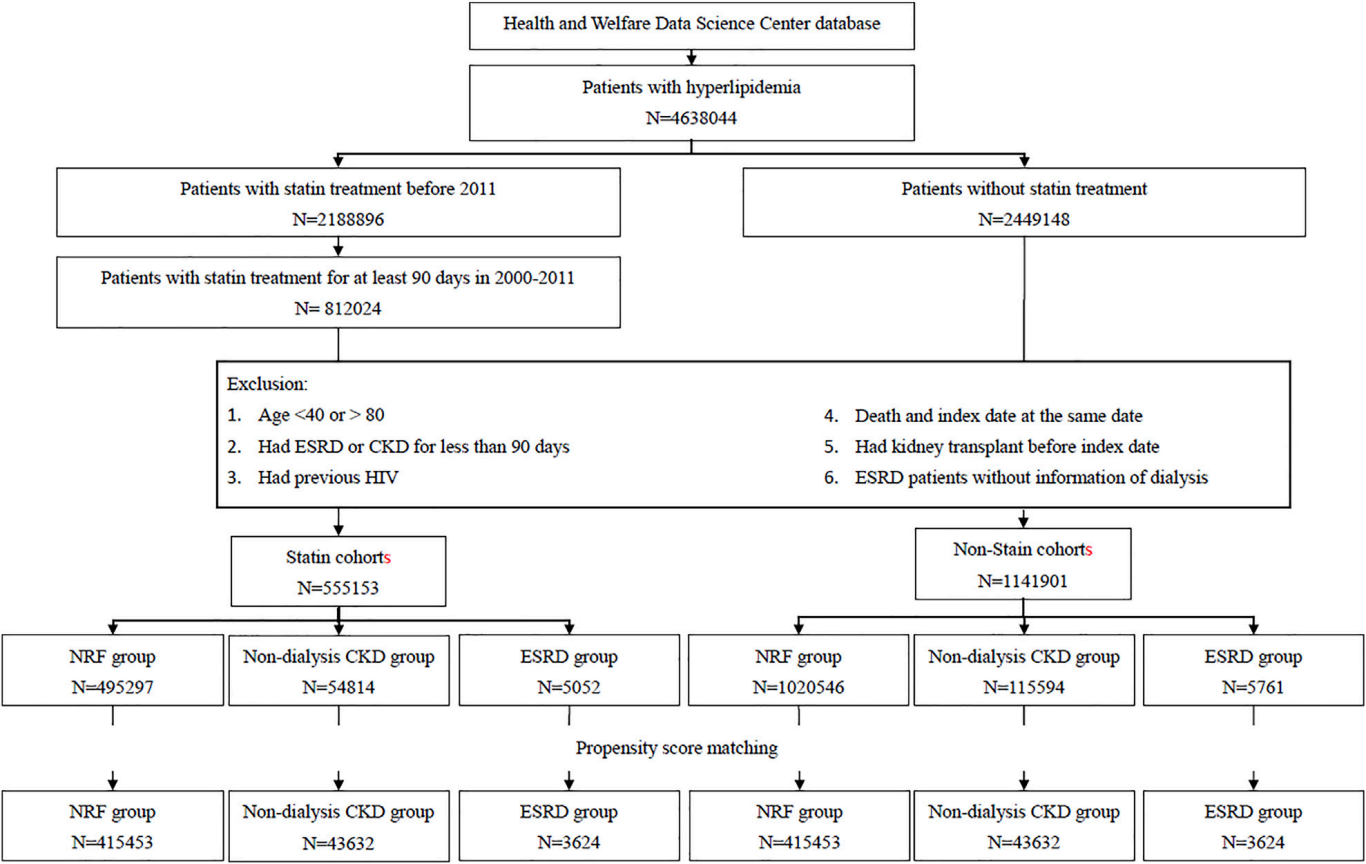
Flow chart for establishing study cohorts.

ST cohort. We identified from the NHIRD 4,638,044 the patients with hyperlipidemia who were newly diagnosed in 2000–2011 in Taiwan. Among these patients, 812,024 patients who had received ST for at least 90 days continuously were selected for the potential cohorts with ST (Figure 1). The 91st day with ST was defined as the index date to address immortal time bias. We excluded patients aged <40 or >80 (n = 71,482), patients with the history of ESRD or CKD for less than 90 days (183,486), patients with HIV (n = 436), patients with kidney transplant history (n = 728), and ESRD patients without dialysis information (n = 739). The remaining 555,153 patients eligible for the ST cohort were stratified into three subcohorts by the renal function: the subcohorts of patients with NRF, with CKD, and with ESRD. Patients without the diagnosis of CKD and ESRD were selected in the NRF group.

Non-ST cohort. From 2,449,148 patients with hyperlipidemia, but without ST, we identified 1,141,901 patients eligible for the non-ST cohort, applying the similar exclusion criteria to the corresponding ST cohort. Likewise, we categorized them into three groups by the renal function: NRF, CKD, and ESRD groups (Figure 1). From each of these three groups, we further randomly selected a non-ST subcohort, as a comparison, corresponding to the specific ST subcohort with a 1:1 ratio, matched by the index date and propensity score (PS). The PS was calculated by multivariable logistic regression, including the variables of age, gender, monthly income, living areas, comorbidities, year hyperlipidemia diagnosed, and index date for NRF group. In the CKD group, we added the CKD diagnosis-year for adjustment. In the ESRD group, we further added ESRD diagnosis-year and HD for adjustments.

### Ethic Statement

All personal identifications in the database had been scrambled and replaced with surrogate numbers before data were released to researchers to protect privacy. This study was approved by the Research Ethics Committee at China Medical University and Hospital (CRREC-107-021) and Ditmanson Medical Foundation Chiayi Christian Hospital (CYCH-IRB-2019063). Because personal privacy was protected from the claims data, the requirement for patient consent was waived. This study was conducted in accordance with the ethics code of the Declaration of Helsinki.

### Outcome and Comorbidity

All study subjects were followed from the index date until death, withdrawal from the insurance, or the end of 2016. The primary outcomes were ACM and three other cause-specific deaths from cancer, heart disease, and septicemia, retrieved from the death registry. Mortalities were compared between patients with and without ST for the three renal groups. Comorbidities including diabetes, hypertension, peripheral arterial occlusive disease, ischemic heart disease, hepatitis B infection, hepatitis C infection, and stroke were considered as covariates. All comorbidities were defined by clinical diagnosis within 2 years before the index date.

### Statistical Analysis

The baseline distributions of the demographic status and comorbidities between each paired subcohort with and without ST in each NRF, CKD, and ESRD group were compared. We calculated the standardized difference of each variable between each paired subcohort. The Kaplan–Meier method calculated and plotted proportional survivals and compared (A) between statin users and nonusers (B) among HS (pravastatin and rosuvastatin) users, LS (including simvastatin, lovastatin, fluvastatin, atorvastatin, cerivastatin, and pitavastatin) users, and nonusers. The ACM rate and each cause specific mortality rate were calculated for each subcohort: the sum of deaths divided by the sum of follow-up years (person-years). We used Cox proportional hazards regression analysis to estimate the ST subcohort-to-non-ST subcohort adjusted hazard ratio (aHR) and a 95% confidence interval (CI) of mortality after controlling for covariates. We further compared the age-specific mortality rates between the ST users and non-users by cause and the renal function group. In order to estimate the short-term impact of ST, we also used a time-dependent Cox regression model to estimate the age-specific ST users to non-user aHRs. All statistical tests were two sided, and the statistical significance was defined as *p*-value <0.05. We used SAS, version 9.4 (SAS Institute, Cary, NC, United States) to conduct data analyses.

## Results

We established three pairs of ST subcohort and non-ST subcohort by the renal function: 415,453 pairs in the NRF group, 43,632 pairs in the CKD group, and 3,624 pairs in the ESRD group (Figure 1). The CKD groups were older with more men than the groups of NRF and ESRD (Table 1). The distributions of income, living area, and comorbidities were similar between each pair of subcohorts. However, the CKD groups and ESRD groups were more likely from lower-income households in central and southern Taiwan and were more prevalent with comorbidities.

**TABLE 1:** Demographic and clinical characteristics of statin cohort and propensity-score matched non-statin cohort in patients with NRF, CKD, and ESRD.

Variable	Normal renal function					Non-dialysis CKD					ESRD				Standardized difference
	Non-statin N = 415,453 n %		Statin N = 415,453 n %		Standardized difference	Non-statin N = 43,632 n %		Statin N = 43,632 n %		Standardized difference	Non-statin N = 3,624 n %		Statin N = 3,624 n %		
Age, mean (SD)	58.7	(10.7)	59.3	(9.94)	0.059	64.2	(10.7)	64.1	(9.99)	0.010	62.2	(10.8)	61.8	(9.89)	0.035
Men, n (%)	199,414	48.0	194,026	46.7	0.026	23,968	54.9	22,680	52.0	0.059	1,690	46.6	1,605	44.3	0.047
Income, NTD															
≤19,200	108,710	26.2	111,341	26.8	0.014	12,795	29.3	12,702	29.1	0.005	1,128	31.1	1,112	30.7	0.010
19,201–21,000	127,763	30.8	127,228	30.6	0.003	14,600	33.5	15,055	34.5	0.022	1,188	32.8	1,220	33.7	0.019
21,001–34,800	83,391	20.1	82,112	19.8	0.008	7,861	18.0	7,648	17.5	0.013	734	20.3	710	19.6	0.017
>34,800	95,589	23.0	94,772	22.8	0.005	8,376	19.2	8,227	18.9	0.009	574	15.8	582	16.1	0.006
Living area															
Northern	18,8952	45.5	192,093	46.2	0.015	16,887	38.7	17,072	39.1	0.009	1,437	39.7	1,440	39.7	0.002
Central	78,779	19.0	77,074	18.6	0.011	9,401	21.6	9,425	21.6	0.001	743	20.5	728	20.1	0.010
Southern	121,452	29.2	118,696	28.6	0.015	14,833	34.0	14,438	33.1	0.019	1,232	34.0	1,231	34.0	0.001
Eastern and offshore islands	26,270	6.32	27,590	6.64	0.013	2,511	5.75	2,697	6.18	0.018	212	5.85	225	6.21	0.015
Comorbidity, n (%)															
DM	125,511	30.2	132,964	32.0	0.039	15,163	34.8	16,721	38.3	0.074	1,887	52.1	1,876	51.8	0.006
Hypertension	181,994	43.8	182,661	44.0	0.003	25,396	58.2	25,146	57.6	0.012	2,495	68.9	2,473	68.2	0.013
PAOD	8,899	2.14	9,254	2.23	0.006	1,516	3.47	1,550	3.55	0.004	314	8.66	326	9.00	0.012
IHD	57,994	14.0	64,611	15.6	0.045	8,688	19.9	9,367	21.5	0.038	1,242	34.3	1,245	34.4	0.002
HBV	7,516	1.81	7,041	1.69	0.009	835	1.91	662	1.52	0.031	119	3.28	118	3.26	0.002
HCV	3,219	0.77	2,744	0.66	0.014	572	1.31	459	1.05	0.024	130	3.59	132	3.64	0.003
Stroke	17,002	4.09	22,993	5.53	0.067	3,050	6.99	3,584	8.21	0.046	291	8.03	299	8.25	0.008
Cancer	15,432	3.71	13,985	3.37	0.019	2,595	5.95	2,133	4.89	0.047	299	8.25	281	7.75	0.018
Hemodialysis											3,243	89.5	3,177	87.7	0.057

Abbreviations: CKD, chronic kidney disease; ESRD, end-stage renal disease; DM, diabetes mellitus; PAOD, peripheral arterial occlusive disease; IHD, ischemic heart disease; HBV, hepatitis B virus infection; HCV, hepatitis C virus infection

**FIGURE. 2:**
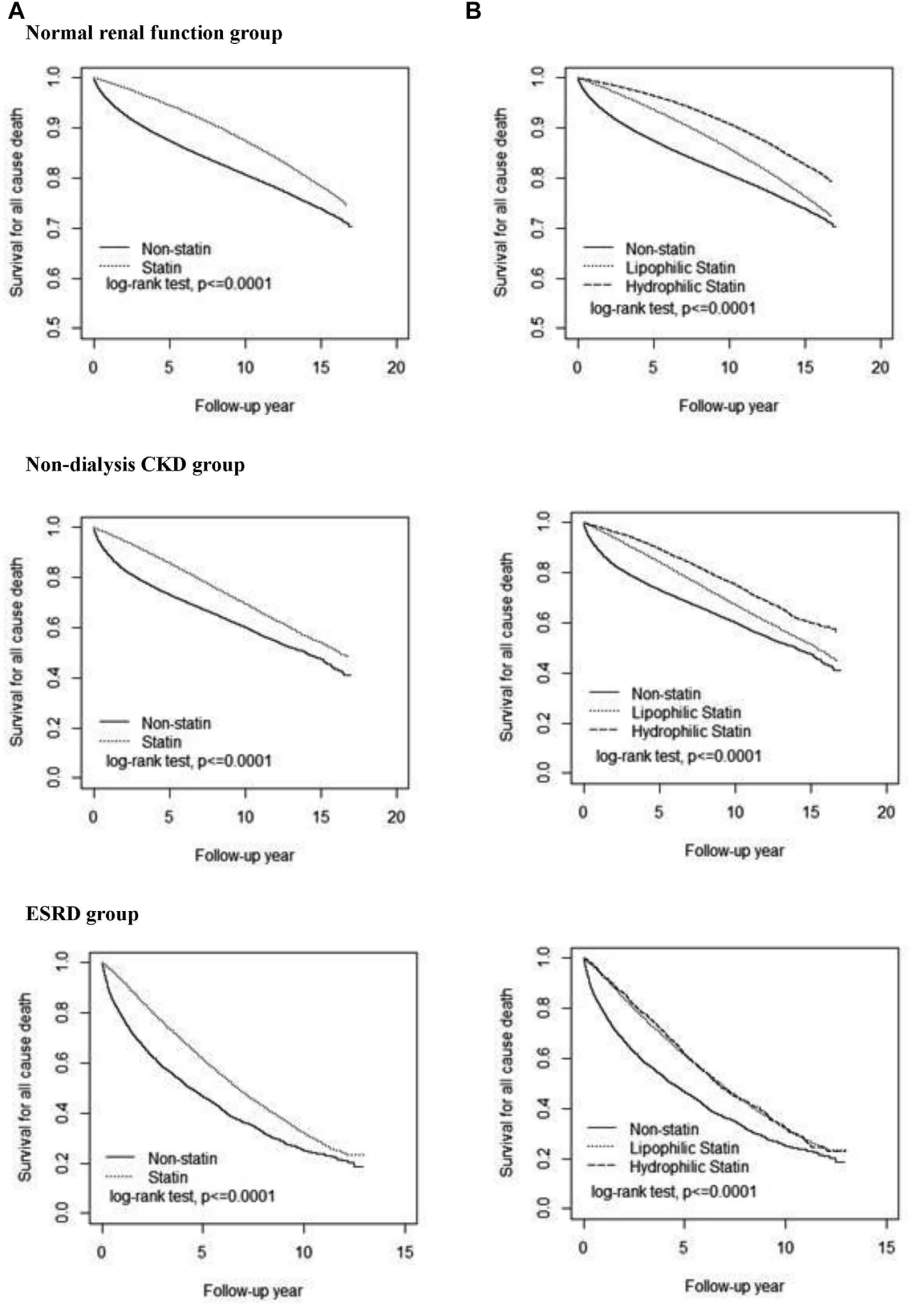
Cumulative survival probability among three renal groups. Comparisons of all-cause death in patients with statin therapy versus non-statin therapy **(A)** and with hydrophilic statin versus lipophilic statin **(B)**. CKD, chronic kidney disease; ESRD, end-stage renal disease.

Kaplan–Meier plots showed that the survival probability was the highest in the NRF group, followed by the CKD group, and the lowest in the ESRD group, higher in the ST subcohort than in the non-ST subcohort in all three renal groups (Figure 2). The survival probability was higher for HS users than for LS users in NRF and CKD groups, but similar to LS users in the ESRD group.

Table 2 shows that ACM and the other three cause-specific mortality rates were substantially greater for patients with ESRD than for those with CKD and NRF in both statin and non-statin subcohorts. The ACM mortality in ESRD patients was 154.6 per 1,000 person-years in the non-ST subcohort and 104.6 per 1,000 person-years in the ST subcohort, with an aHR of 0.68 (95% CI = 0.64–0.72) for the ST users. The corresponding ACM rates in CKD patients were 56.4 versus 35.9 per 1,000 person-years, with an aHR of 0.64 (95% CI = 0.62–0.65) for the ST users. The corresponding ACM rates in the NRF group were 22.6 versus 13.9 per 1,000 person-years, with an aHR of 0.62 (95% CI = 0.60–0.63) for the ST users. The mortality rates of hyperlipidemic patients with cancer, heart disease, and septicemia were all lower for ST users than for non-ST users in the three renal groups. However, the ST users to non-users aHR of death from heart disease were not significant for ESRD patients.

**TABLE 2:** Mortality compared between statin users and non-users by cause and renal group.

Outcome	Non-statin			Statin			aHR (95%CI)
	Event no.	PYs	Rate, per 1,000 PYs	Event no.	PYs	Rate, per 1,000 PYs	
All-cause death							
Normal renal function	73,560	3,249,626	22.6	54,517	3,916,082	13.9	0.62 (0.60–0.63)^b^
Non-dialysis CKD	15,377	272,621	56.4	11,642	324,339	35.9	0.64 (0.62–0.65)^b^
ESRD	2,278	14,734	154.6	2,186	20,904	104.6	0.68 (0.64–0.72)^b^
p for trend			<0.0001			<0.0001	
Cancer death							
Normal renal function	23,821	3,249,626	7.33	15,868	3,916,082	4.05	0.56 (0.55–0.57)^b^
Non-dialysis CKD	3,226	272,621	11.8	2,078	324,339	6.41	0.54 (0.51–0.57)^b^
ESRD	231	14,734	15.7	204	20,904	9.76	0.63 (0.52–0.75)^b^
p for trend			<0.0001			<0.0001	
Heart disease death							
Normal renal function	7,214	3,249,626	2.22	8,196	3,916,082	2.09	0.94 (0.91–0.97)^b^
Non-dialysis CKD	1,780	272,621	6.53	1,825	324,339	5.63	0.86 (0.80–0.91)^b^
ESRD	228	14,734	15.5	291	20,904	13.9	0.89 (0.74–1.06)
p for trend			<0.0001			<0.0001	
Septicemia death							
Normal renal function	1,100	3,249,626	0.37	1,033	3,916,082	0.26	0.75 (0.69–0.82)^b^
Non-dialysis CKD	307	272,621	1.13	308	324,339	0.95	0.83 (0.71–0.97)^a^
ESRD	73	14,734	4.95	71	20,904	3.40	0.69 (0.50–0.95)^a^
p for trend			<0.0001			<0.0001	

CKD, chronic kidney disease; ESRD, end-stage renal disease; PYs, person-year; aHR, adjusted hazard ratio and CI, confidence interval, estimated controlling for age, gender, and all comorbidities.

a *p* < 0.05

b *p* < 0.001

The results from the time-dependent Cox regression analysis showed that the short-term impact of ST was also associated with the reduced aHRs of deaths, which is also significant for ACM and cancer deaths (Supplementary Table S2). The aHR of ACM for ST users with an ESRD of 40–59 years old was 0.33 (95% CI = 0.28–0.40), which was reduced slightly to 0.31 (95% CI = 0.26–0.37) for 70–80 years old. However, the short-term effect of ST on deaths from heart disease and septicemia in 40–59 years old with NRF was not significant. The treatment effects of ST were also not significant on deaths from heart disease for older patients with ESRD.

**FIGURE. 3:**
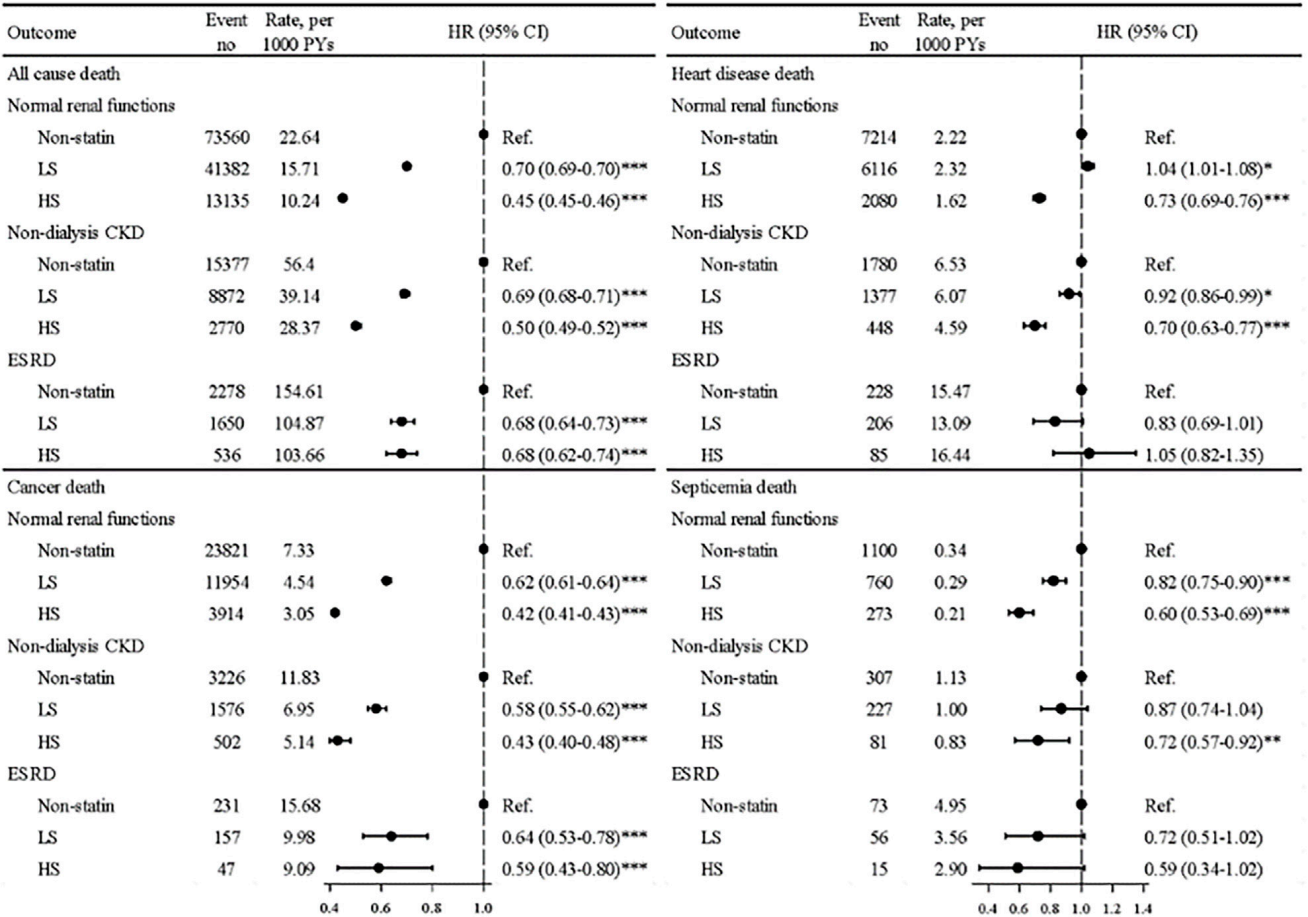
Mortality rates from all causes, cancer, heart disease, and septicemia and aHRs in patients using hydrophilic statins, lipophilic statins, and nonusers among three renal groups.

Figure 3 shows, in general, that the ST treatment effectiveness was superior in HS users than in LS users, with lower mortality rates from most types of death compared with nonusers. All aHRs were significant for deaths from all causes and from heart disease. For ACM in the ESRD group, the mortality rate for HS users was 51 per 1,000 person-years lower than that for non-statin patients, with an aHR of 0.68 (95% CI = 0.62–0.74). For patients with NRF or CKD, the hazards of deaths from heart disease were significantly reduced in HS users but not in LS users. The ST treatment effectiveness was superior in HS users than in LS users for reducing deaths from septicemia in the NRF and CKD groups.

## Discussion

### Findings

Our data showed that hyperlipidemic dialysis patients receiving ST were significantly associated with reduced mortality hazards for 32% from ACM, for 37% from cancer, and for 31% from septicemia-related events, whereas the benefit of reducing deaths from heart disease was less obvious (Table 2). The dialysis patients receiving HS or LS were also associated with reduced risks of deaths from ACM and cancer. The beneficial effects on deaths from ACM were similar for using HS and LS, whereas the reduction effect on deaths from cancer was slightly greater for using HS than using LS (Figure 2 and Figure 3). Our age-specific data showed that the ACM rate increased with age (Supplementary Table S1). However, statin users’ to non-users’ aHR of deaths from ACM and from other causes tended to decrease with age. These data suggested that the treatment effectiveness of statins might exert a greater impact in the death risk reduction for the elderly than for youngsters.

A simulated 4D trial of ST on diabetic ESRD patients also reported significant cardiovascular benefits for those with the therapy (Chan et al., 2010). This simulated observational trial may help in providing evidence to develop potential studies on treatment effectiveness. Several recent studies using real-world data have also reported the ST benefit in the associations with reduced ACM for patients undergoing HD (De Rango et al., 2016; Chung et al., 2017; Kim et al., 2018; Jung et al., 2020). An Italian study comparing the ACM risk between HD patients with ST (n = 127) and without ST (n = 232) found that ST reduced the risk for nearly 50%, 5 years after a vascular access operation (De Rango et al., 2016). A population-based study in Taiwan comparing 790 pairs of HD patients with and without ST after acute MI episodes found the ACM risk reduced for 24%–30% in ST users (Chung et al., 2017). A Korean population-based study found that ST was associated with 46% reduced risk of composite cardiovascular events, including ACM (Kim et al., 2018). Another Korean population-based observational study revealed that in adult HD patients, statin and ezetimibe combined treatment was associated with a halved risk of ACM (Jung et al., 2020). These reports support our research findings relating to ACM in dialysis patients.

The increasing noncardiovascular deaths in ESRD patients in recent years, especially in the elderly, have attracted attention (Den Hoedt et al., 2013; Vogelzang et al., 2015; Steenkamp et al., 2018). In Taiwan, infection and cancer are the major non-cardiovascular causes for the cost of admission healthcare for dialysis patients (Hsu, 2020). Studies have reported that statins possess anti-inflammatory capacities and ST was associated with improved survival in patients with Gram-positive or Gram-negative bacterial infections and sepsis (Ou et al., 2014; Mehl et al., 2015; Ouellette et al., 2015; Shrestha et al., 2016; Caffrey et al., 2017). A propensity-score matched data analysis found that the high-potency statin was associated with a lower risk of sepsis-related mortality than low-potency statin uses (Ou et al., 2014). A meta-analysis based on six cohort studies with 7,553 patients of bacterial infection without renal dysfunction showed that ST could reduce the mortality risk for nearly 30% (Shrestha et al., 2016), although, in a systemic study, Collins et al. have a different perspective and commented the absence of evidence that ST provided clinical benefits in non-cardiovascular health outcomes such as cancer or infection (Collins et al., 2016).

However, recent studies provided more evidence of the clinical benefits of ST in noncardiovascular conditions. For cancer patients, evidence has revealed that adherence to ST is essential to achieve the beneficial effects (Lee et al., 2020; Feng and Qin, 2021; Okada et al., 2021). A Korean population-based retrospective cohort study found that the treatment effectiveness of ST relied on good statin adherence (Lee et al., 2020); poor adherence is associated with an increased risk of deaths from cancer or CVD, with the HR of nearly 1.3 compared to patients with good adherence. An Australian Cancer database study reported that good adherence to lipid-lowering medications was associated with reduced mortality from breast cancer, colorectal cancer, or melanoma (Feng and Qin, 2021). A Japanese study with 2,536 patients of type 2 diabetes reported that ST was associated with a 40% reduction in cancer mortality (Okada et al., 2021).

Statins are medicines effective in inhibiting the enzyme HMG-CoA reductase and the following mevalonate/isoprenoid pathways, exerting pleiotropic effects but widely used as lipid-lowering medications (Ahmadi et al., 2017). With anti-inflammatory effects, statins can inhibit the production of TNFa and IL-6 by mast cells, and activate the endothelial cells, exerting antioxidant effects (Nseir et al., 2010). With pleiotropic effects, the anti-inflammatory and antiproliferative properties of statin might partially explain the clinical benefits (Barbalata et al., 2020). Besides, prolific basic lab researches have provided strong evidences supporting the anti-cancer effects of statins, which may block diverse carcinogenic pathways (Bonsu et al., 2016). These might be the proposed mechanism of protection of statins for the reduction of all-cause mortality and non-CVD mortality.

A previous research comparing the mortality associated with HS and LS was conducted mainly in non-CKD patients with CVDs, with inconsistent findings. One meta-analysis reported that the LS medication is associated with reduced ACM and cardiovascular mortality in patients with congestive heart failure (Bonsu et al., 2016). A meta-analysis and a randomized trial reported the similar treatment effectiveness of the medications of HS and LS on major adverse cardiac events (Izawa et al., 2015; Bytyçi et al., 2017). Another PS-matched retrospective cohort study using the insurance data of Taiwan found a greater ACM risk reduction in patients receiving HS than in LS users for 35% (Chung et al., 2018), which is similar to our finding in the NRF group.

It is also noteworthy in our study that the treatment effectiveness of ST in reducing deaths from heart disease was not significant for the ESRD group, whereas in NRF and CKD patients, the ST effectiveness was significant by providing nearly a 30% risk reduction. These findings in non-ESRD group are agreeable to current literature (Palmer et al., 2014). In patients with ESRD, there is a complicated cardiovascular pathogenesis process, involving vascular calcification in both intimal and medial layers, sympathetic hyperactivity, and myocardial fibrosis, leading to a complicated cardiovascular scenario and poor response to a certain therapy that is helpful to non-dialysis groups (Santoro and Mandreoli, 2014; Laucyte-Cibulskiene et al., 2018).

### Limitations

There are several limitations in our research. First, this is an observational study retrieving the information of patients from a secondary database. Due to the characteristics of the database, certain key information such as lab data and a personal lifestyle of smoking, dietary habits, and exercise was unavailable for adjustment in the data analysis. In order to reduce potential bias and the confounding effects of these factors, we addressed immortal time bias and conducted a time-variable model analysis. We used PS matching to establish study cohorts to reduce the personal variations and effects of comorbidities, even though residual confounding effects may be present since we did not adjust for lab data. Furthermore, the information on the exact patient medication compliances was unavailable for adjustment as well. Second, our research was conducted in a Taiwanese population-based setting; the generalizability to other ethnical cohorts or healthcare systems might be limited. Lastly, we used a death registry to retrieve the data of heart disease death as a proxy for cardiovascular death. Although the death registry provides highly accurate information about the causes of death, heart disease deaths included some deaths other than the scenario of major cardiovascular death, which might contribute a bias in the risk measurement.

### Strength

There are strengths in our study. The sample sizes of the study cohorts were very large, allowing us to conduct a comprehensive stratification analysis and adjustment without jeopardizing the power of analysis. Our study investigated the effectiveness of ST on the association with mortality risks in three different renal groups. The mortality risk reductions in patients with NRF and CKD were consistent with current evidences (Palmer et al., 2014; Lin et al., 2020). The sample sizes of most previous studies were likely not large enough to include patients with ESRD for analysis. To the best of our knowledge, no previous study has ever evaluated simultaneously the risks of death not only from all causes but also from cancer, heart disease, and septicemia. Septicemia deaths are relatively rare compared to other causes. Our sample is large and enables us to observe the effectiveness of ST in reducing the mortality from septicemia, even for patients with ESRD. Previous studies rarely compared the treatment effectiveness between HS and LS for all these causes. Our study demonstrated that the effectiveness of HS might be superior than LS. However, our reports shed light on further studies to investigate the mechanisms leading to the variety of effectiveness of ST in patients with and without renal dysfunction.

The findings from the present study could derive clinical implications. First, the absence of a significant clinical benefit from ST in reducing the mortality from heart disease in the ESRD group indicates the complicated cardiovascular scenario in ESRD and potential harmful effects associated with ST. Both factors call for the need for novel and more promising lipid-lowering medications, such as proprotein convertase subtilisin/kexin Type 9 (PCSK9) inhibitors. We confirmed the efficacies and indications of ST as an effective lipid-lowering medication for hyperlipidemic dialysis patients, preferably in the older population. Clinicians might need to have a thorough understanding on statins, especially in caring for dialysis patients with effective statin products. Second, the high need for an effective treatment for ESRD calls for more studies. However, the research with an RCT is under financial constraints. Observational studies help to provide clues for further evidence. Our findings may pave the way for investigating the evidence with which the relationship between statins and ESRD patients might be delineated.

## Conclusion

Patients with ESRD receiving ST are at reduced risks for deaths from all causes, cancer, heart disease, and septicemia but not significant for deaths from the heart disease. The medication with HS is likely associated with better death risk reduction than the medication of LS. The age-specific analysis results suggest that the ST is appropriate for the elderly patients. Further large-scale prospective trials are needed to confirm our preliminary findings.

## Data Availability

The original contributions presented in the study are included in the article/Supplementary Material, further inquiries can be directed to the corresponding author.
