# Supplementary material for: Statin Therapy for Hyperlipidemic Patients With Chronic Kidney Disease and End-Stage Renal Disease: A Retrospective Cohort Study Based on 925,418 Adults in Taiwan
Source: Front Pharmacol. 2022 Mar 4;13:815882. doi: 10.3389/fphar.2022.815882 (PMC8930832; doi:10.3389/fphar.2022.815882)
Supplement: Supplementary file 1 [file DataSheet1.docx]

Supplementary Material

| **Table S1. Mortality rate by cause and hazard ratio of death for CKD and ESRD groups compared to normal renal function group** | | | | | | | |
| --- | --- | --- | --- | --- | --- | --- | --- |
| **Outcome** | **Event no** | **PYs** | **Rate, per 1000 PYs** | **Crude HR (95% CI)** | **p** | **Adjusted HR (95% CI)** | **p** |
| All cause death |  |  |  |  |  |  |  |
| Normal renal functions | 71911 | 4759110 | 15.11 | Ref. |  | Ref. |  |
| Non-dialysis CKD | 15010 | 416933 | 36.00 | 2.47 (2.42-2.51) | <0.0001 | 1.70 (1.67-1.73) | <0.0001 |
| ESRD | 2986 | 30665 | 97.38 | 7.22 (6.96-7.49) | <0.0001 | 7.83 (7.55-8.13) | <0.0001 |
| Cancer death |  |  |  |  |  |  |  |
| Normal renal functions | 31760 | 4759110 | 6.67 | Ref. |  | Ref. |  |
| Non-dialysis CKD | 6647 | 416933 | 15.94 | 2.48 (2.41-2.54) | <0.0001 | 1.71 (1.67-1.76) | <0.0001 |
| ESRD | 1379 | 30665 | 44.97 | 7.56 (7.16-7.98) | <0.0001 | 8.22 (7.78-8.68) | <0.0001 |
| Heart disease death |  |  |  |  |  |  |  |
| Normal renal functions | 11876 | 4759110 | 2.50 | Ref. |  | Ref. |  |
| Non-dialysis CKD | 2579 | 416933 | 6.19 | 2.57 (2.46-2.68) | <0.0001 | 1.77 (1.70-1.85) | <0.0001 |
| ESRD | 527 | 30665 | 17.19 | 7.76 (7.11-8.47) | <0.0001 | 8.37 (7.66-9.15) | <0.0001 |
| Septicemia death |  |  |  |  |  |  |  |
| Normal renal functions | 1947 | 4759110 | 0.41 | Ref. |  | Ref. |  |
| Non-dialysis CKD | 402 | 416933 | 0.96 | 2.45 (2.20-2.73) | <0.0001 | 1.68 (1.50-1.87) | <0.0001 |
| ESRD | 85 | 30665 | 2.77 | 7.74 (6.22-9.63) | <0.0001 | 8.21 (6.58-10.2) | <0.0001 |
| aHR: adjusted hazard ratio; CI: confidence interval, estimated controlling for age, gender and all comorbidities; CKD:chronic kidney disease; ESRD: end stage renal disease; PYs: person-year. | | | | | | | |

| **Table S2. Age-specific mortality by type of death, renal subgroup and statin cohort to non-statin cohort adjusted hazard ratio of death.** | | | | | | | | |
| --- | --- | --- | --- | --- | --- | --- | --- | --- |
|  | **Non-statin** | | | **Statin** | | |  | |
| **Outcome** | **Event no** | **PYs** | **Rate, per 1000 PYs** | **Event no** | **PYs** | **Rate, per 1000 PYs** | **aHR (95% CI)** | **p** |
| All cause death |  |  |  |  |  |  |  |  |
| Normal renal functions |  |  |  |  |  |  |  |  |
| 40-59 | 19230 | 1991370 | 9.66 | 13370 | 2144472 | 6.23 | 0.65 (0.64-0.67) | <0.0001 |
| 60-69 | 19439 | 757774 | 25.65 | 16752 | 116904 | 15.00 | 0.58 (0.56-0.59) | <0.0001 |
| 70-80 | 34891 | 500483 | 69.71 | 24395 | 654706 | 37.26 | 0.52 (0.51-0.53) | <0.0001 |
| Non-dialysis CKD |  |  |  |  |  |  |  |  |
| 40-59 | 2058 | 108868 | 18.90 | 1500 | 112444 | 13.34 | 0.71 (0.66-0.76) | <0.0001 |
| 60-69 | 3650 | 77448 | 47.13 | 3207 | 105246 | 30.47 | 0.64 (0.61-0.67) | <0.0001 |
| 70-80 | 9669 | 86305 | 112.03 | 6935 | 106649 | 65.03 | 0.58 (0.56-0.59) | <0.0001 |
| ESRD |  |  |  |  |  |  |  |  |
| 40-59 | 670 | 7502 | 89.31 | 664 | 10384 | 63.94 | 0.71 (0.64-0.80) | <0.0001 |
| 60-69 | 692 | 3905 | 177.19 | 748 | 6218 | 120.30 | 0.68 (0.61-0.75) | <0.0001 |
| 70-80 | 916 | 3327 | 275.36 | 774 | 4302 | 179.90 | 0.66 (0.60-0.72) | <0.0001 |
| Cancer death |  |  |  |  |  |  |  |  |
| Normal renal functions |  |  |  |  |  |  |  |  |
| 40-59 | 6931 | 1991370 | 3.48 | 4528 | 2144472 | 2.11 | 0.60 (0.58-0.63) | <0.0001 |
| 60-69 | 7343 | 757774 | 9.69 | 5323 | 116904 | 4.77 | 0.49 (0.47-0.50) | <0.0001 |
| 70-80 | 9547 | 500483 | 19.08 | 6017 | 654706 | 9.19 | 0.49 (0.47-0.50) | <0.0001 |
| Non-dialysis CKD |  |  |  |  |  |  |  |  |
| 40-59 | 500 | 108868 | 4.59 | 300 | 112444 | 2.67 | 0.57 (0.49-0.66) | <0.0001 |
| 60-69 | 911 | 77448 | 11.76 | 682 | 105246 | 6.48 | 0.54 (0.49-0.60) | <0.0001 |
| 70-80 | 1815 | 86305 | 21.03 | 1096 | 106649 | 10.28 | 0.49 (0.45-0.52) | <0.0001 |
| ESRD |  |  |  |  |  |  |  |  |
| 40-59 | 67 | 7502 | 8.93 | 63 | 10384 | 6.07 | 0.67 (0.48-0.94) | 0.019 |
| 60-69 | 77 | 3905 | 19.72 | 68 | 6218 | 10.94 | 0.56 (0.41-0.77) | 0.0004 |
| 70-80 | 87 | 3327 | 26.15 | 73 | 4302 | 16.97 | 0.65 (0.48-0.88) | 0.006 |
| Heart disease death |  |  |  |  |  |  |  |  |
| Normal renal functions |  |  |  |  |  |  |  |  |
| 40-59 | 1510 | 1991370 | 0.76 | 2084 | 2144472 | 0.97 | 1.28 (1.20-1.37) | <0.0001 |
| 60-69 | 1790 | 757774 | 2.36 | 2377 | 116904 | 2.13 | 0.87 (0.82-0.93) | <0.0001 |
| 70-80 | 3914 | 500483 | 7.82 | 3735 | 654706 | 5.70 | 0.71 (0.67-0.74) | <0.0001 |
| Non-dialysis CKD |  |  |  |  |  |  |  |  |
| 40-59 | 170 | 108868 | 1.56 | 237 | 112444 | 2.11 | 1.32 (1.08-1.61) | 0.006 |
| 60-69 | 377 | 77448 | 4.87 | 475 | 105246 | 4.51 | 0.91 (0.80-1.04) | 0.172 |
| 70-80 | 1233 | 86305 | 14.29 | 1113 | 106649 | 10.44 | 0.72 (0.67-0.78) | <0.0001 |
| ESRD |  |  |  |  |  |  |  |  |
| 40-59 | 79 | 7502 | 10.53 | 86 | 10384 | 8.28 | 0.75 (0.55-1.02) | 0.069 |
| 60-69 | 67 | 3905 | 17.16 | 106 | 6218 | 17.05 | 0.96 (0.71-1.31) | 0.816 |
| 70-80 | 82 | 3327 | 24.65 | 99 | 4302 | 23.01 | 0.94 (0.70-1.26) | 0.661 |
| Septicemia death |  |  |  |  |  |  |  |  |
| Normal renal functions |  |  |  |  |  |  |  |  |
| 40-59 | 204 | 1991370 | 0.10 | 164 | 2144472 | 0.08 | 0.74 (0.60-0.90) | 0.003 |
| 60-69 | 257 | 757774 | 0.34 | 290 | 116904 | 0.26 | 0.70 (0.59-0.83) | <0.0001 |
| 70-80 | 639 | 500483 | 1.28 | 579 | 654706 | 0.88 | 0.63 (0.56-0.70) | <0.0001 |
| Non-dialysis CKD |  |  |  |  |  |  |  |  |
| 40-59 | 28 | 108868 | 0.26 | 35 | 112444 | 0.31 | 1.22 (0.74-1.99) | 0.435 |
| 60-69 | 70 | 77448 | 0.90 | 68 | 105246 | 0.65 | 0.69 (0.49-0.96) | 0.029 |
| 70-80 | 209 | 86305 | 2.42 | 205 | 106649 | 1.92 | 0.77 (0.63-0.92) | 0.006 |
| ESRD |  |  |  |  |  |  |  |  |
| 40-59 | 22 | 7502 | 2.93 | 21 | 10384 | 2.02 | 0.70 (0.39-1.27) | 0.244 |
| 60-69 | 19 | 3905 | 4.87 | 22 | 6218 | 3.54 | 0.71 (0.40-1.29) | 0.264 |
| 70-80 | 32 | 3327 | 9.62 | 28 | 4302 | 6.51 | 0.68 (0.41-1.12) | 0.125 |
| aHR: adjusted hazard ratio; CI: confidence interval, estimated controlling for age, gender and all comorbidities; CKD: chronic kidney disease; ESRD: end stage renal disease; PYs: person-year. | | | | | | | | |

| **Table S3. Adjusted hazard ratio of death between statin and non-statin cohort in three groups in Cox model with time-dependent covariates** | | | | | | | | | |
| --- | --- | --- | --- | --- | --- | --- | --- | --- | --- |
|  | All | | Age 40-59 | | Age 60-69 | | Age 70-80 | |  |
| Outcome | aHR (95% CI) | p-value | aHR (95% CI) | p-value | aHR (95% CI) | p-value | aHR (95% CI) | p-value |  |
| All cause death |  |  |  |  |  |  |  |  |  |
| Normal renal functions | 0.28 (0.26-0.30) | <0.0001 | 0.37 (0.32-0.42) | <0.0001 | 0.27 (0.24-0.31) | <0.0001 | 0.23 (0.21-0.26) | <0.0001 |  |
| Non-dialysis CKD | 0.27 (0.26-0.28) | <0.0001 | 0.34 (0.30-0.38) | <0.0001 | 0.29 (0.27-0.32) | <0.0001 | 0.25 (0.24-0.27) | <0.0001 |  |
| ESRD | 0.32 (0.28-0.35) | <0.0001 | 0.33 (0.28-0.40) | <0.0001 | 0.34 (0.28-0.40) | <0.0001 | 0.31 (0.26-0.37) | <0.0001 |  |
| Cancer death |  |  |  |  |  |  |  |  |  |
| Normal renal functions | 0.10 (0.08-0.13) | <0.0001 | 0.17 (0.13-0.23) | <0.0001 | 0.07 (0.05-0.10) | <0.0001 | 0.08 (0.06-0.12) | <0.0001 |  |
| Non-dialysis CKD | 0.11 (0.10-0.13) | <0.0001 | 0.09 (0.05-0.14) | <0.0001 | 0.12 (0.09-0.16) | <0.0001 | 0.12 (0.10-0.15) | <0.0001 |  |
| ESRD | 0.22 (0.15-0.33) | <0.0001 | 0.19 (0.09-0.40) | <0.0001 | 0.20 (0.10-0.41) | <0.0001 | 0.30 (0.16-0.55) | <0.0001 |  |
| Heart disease death |  |  |  |  |  |  |  |  |  |
| Normal renal functions | 0.57 (0.49-0.67) | <0.0001 | 0.80 (0.60-1.07) | 0.127 | 0.60 (0.46-0.79) | <0.0001 | 0.45 (0.35-0.57) | <0.0001 |  |
| Non-dialysis CKD | 0.48 (0.43-0.54) | <0.0001 | 0.70 (0.53-0.91) | 0.008 | 0.58 (0.49-0.71) | <0.0001 | 0.43 (0.37-0.49) | <0.0001 |  |
| ESRD | 0.61 (0.47-0.78) | <0.0001 | 0.44 (0.27-0.71) | 0.001 | 0.79 (0.54-1.17) | 0.247 | 0.69 (0.45-1.05) | 0.085 |  |
| Septicemia death |  |  |  |  |  |  |  |  |  |
| Normal renal functions | 0.31 (0.19-0.52) | <0.0001 | 0.54 (0.19-1.54) | 0.249 | 0.43 (0.18-1.00) | 0.051 | 0.20 (0.09-0.45) | <0.0001 |  |
| Non-dialysis CKD | 0.24 (0.17-0.34) | <0.0001 | 0.14 (0.03-0.56) | 0.006 | 0.29 (0.15-0.53) | <0.0001 | 0.25 (0.17-0.39) | <0.0001 |  |
| ESRD | 0.19 (0.09-0.40) | <0.0001 | 0.27 (0.08-0.87) | 0.028 | 0.09 (0.01-0.64) | 0.016 | 0.23 (0.07-0.73) | 0.013 |  |
| aHR: adjusted hazard ratio; CI: confidence interval estimated controlling for age, gender and all comorbidity. | | | | | | | | | |
